# Seronegative Autoimmune Hepatitis: A Rare Manifestation of COVID-19

**DOI:** 10.7759/cureus.45688

**Published:** 2023-09-21

**Authors:** Hwewon E Lee, Julia Zhang, Alyeesha B Wilhelm, Heather L Stevenson, Sheharyar Merwat

**Affiliations:** 1 Department of Internal Medicine, University of Texas Medical Branch at Galveston, Galveston, USA; 2 Department of Gastroenterology and Hepatology, University of Texas Medical Branch at Galveston, Galveston, USA; 3 Department of Pathology, University of Texas Medical Branch at Galveston, Galveston, USA

**Keywords:** autoimmune hepatitis, liver disease, covid-19 and liver, liver, seronegative autoimmune hepatitis

## Abstract

Severe acute respiratory syndrome coronavirus 2 (SARS-CoV-2), the virus responsible for the coronavirus pandemic in 2019, commonly causes hepatic dysfunction. Liver injury ranges from mildly elevated liver enzymes to fulminant liver failure. Interestingly, there are cases that suggest a relationship between autoimmune hepatitis (AIH) in patients who either contracted coronavirus disease in 2019 (COVID-19) or were vaccinated against severe acute respiratory syndrome coronavirus 2 (SARS-CoV-2). We present a case of a 39-year-old female without a significant past medical history who presented with two weeks of jaundice, abdominal pain, nausea, and diarrhea. She had significantly elevated liver enzymes and conjugated hyperbilirubinemia. She also tested positive for SARS-CoV-2 but denied any respiratory symptoms; her vaccination status was up to date. She denied taking hepatotoxic agents, and the workup was negative for acute viral hepatitis. The F-actin antibody level was 22 units, but serum immunoglobulin (IgG), anti-nuclear (ANA), anti-smooth muscle, anti-mitochondrial, anti-liver/kidney microsomal-1, anti-soluble liver antigen, and anti-neutrophil cytoplasmic antibodies levels were not elevated. Computerized tomography of the abdomen and pelvis revealed hepatic hemangiomas. Eventually, a liver biopsy was performed, and histology showed active lymphoplasmacytic hepatitis with prominent regenerative changes and areas of confluent necrosis. The histologic findings, along with the patient's clinical course, were suggestive of autoimmune hepatitis. The patient was started on systemic steroids with an improvement of abdominal pain and jaundice, as well as an improvement of her liver chemical profile. She was discharged with plans for hepatology clinic follow-up. Here, we present a rare case of seronegative AIH in a patient with a recent COVID-19 infection and discuss the potential underlying mechanism. We call for further investigation into the relationship between autoimmune dysfunction and COVID-19, as well as the pathophysiology behind it. Analyzing how the virus causes autoimmune dysfunction may allow clinicians to more effectively treat patients suffering from sequelae of COVID-19 infection, and it is important not to exclude autoimmune hepatitis from the differential based on the initial autoimmune workup.

## Introduction

Following the emergence of the coronavirus disease in 2019 (COVID-19), there have been numerous studies looking into the pathophysiology, manifestations, and long-term effects of this Orthocoronavirinae subfamily. The virus mainly affects the upper and lower respiratory tracts in humans, but just about any organ can be affected, including the liver. About 14-53% of patients with COVID-19 have complications of hepatic dysfunction, especially if they have underlying comorbidities [1]. In these patients with hepatic dysfunction, there are a few reports highlighting COVID-19 infection and/or vaccination leading to the development of autoimmune hepatitis (AIH) [2-5]. AIH is usually associated with circulating autoantibodies such as anti-nuclear antibody (ANA) and anti-smooth muscle antibody, but 10-20% of AIH patients do not show any serologic markers, which is also known as seronegative autoimmune hepatitis (SAH) [6]. There are only a handful of reported cases of seronegative AIH following the COVID-19 infection in the United States.

## Case presentation

A 39-year-old, otherwise healthy female was admitted to the hospital for two weeks of nausea, abdominal pain, and jaundice. She denied taking any hepatotoxic agents and did not have a significant alcohol or drug use history. Family history was notable for the patient’s mother who has rheumatoid arthritis.

The patient’s admission labs were significant for total bilirubin 7.6 mg/dL (conjugated bilirubin 4.2 mg/dL, unconjugated bilirubin 1.4 mg/dL), alkaline phosphatase 244 U/L, alanine transaminase 1057 U/L, and aspartate transaminase 1270 U/L. Hemoglobin was 12.1 g/dL, white blood cell count 6.24/uL, platelets 275/uL, albumin 3.6 g/dL, and international normalized ratio 1.3. The workup for causes of hepatitis, including serologies for acute viral hepatitis, alpha-1-antitrypsin, and ceruloplasmin, was unremarkable. The F-actin antibody level was 22 units, but anti-smooth muscle and ANA were negative. Serum immunoglobulin G (IgG) was less than 1800 mg/dL, and anti-mitochondrial, anti-soluble liver antigen, anti-liver/kidney microsomal-1, and anti-neutrophil cytoplasmic antibodies were unremarkable. The acetaminophen level, salicylate level, and urine toxicology were unremarkable as well. She tested positive for COVID-19 but denied any respiratory symptoms, and she was up to date with her vaccination, which was six months ago.

**Figure 1:**
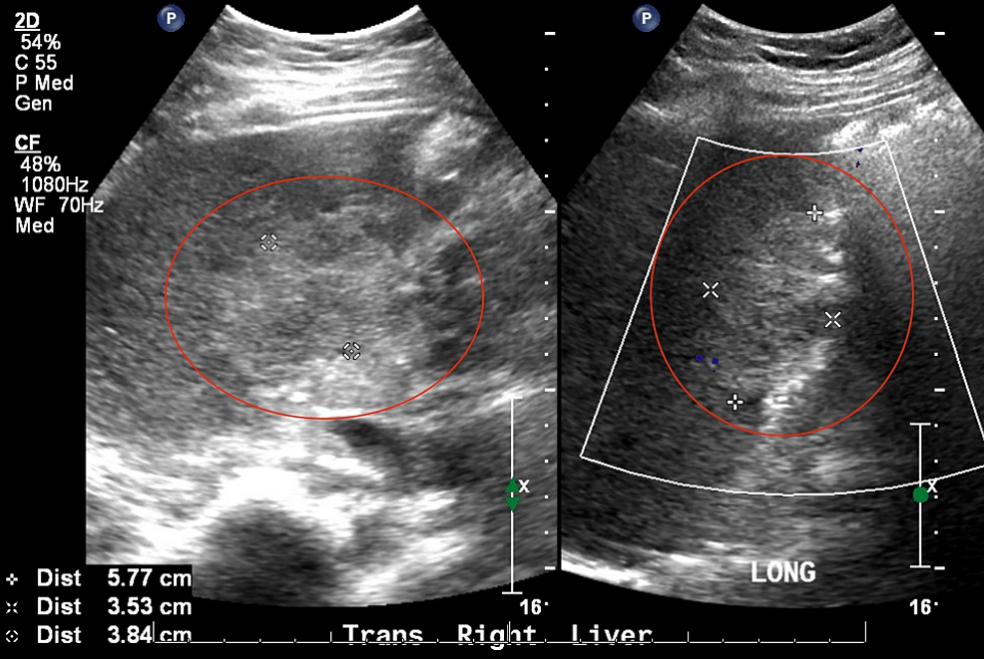
Abdominal ultrasound with Doppler showed a large right hyper-echoic hepatic lesion (red circles) and mildly increased overall hepatic parenchymal echogenicity.

Right upper quadrant ultrasound with Doppler showed a patent portal vein with mildly increased hepatic parenchymal echogenicity and two hyper-echoic hepatic lesions (Figure 1).

**Figure 2:**
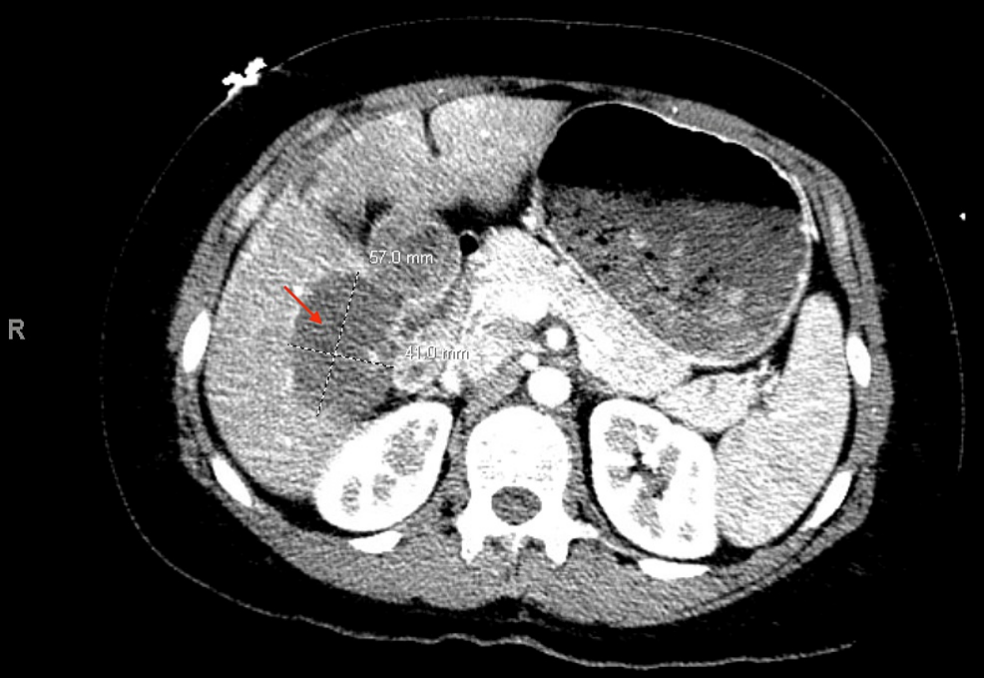
Computerized topography of the abdomen and pelvis showing an indeterminate, wedge-like geographic focus of severe hypoattenuation with internal hyperattenuating foci in the subcapsular right hepatic lobe, suggestive of hepatic hemangioma or hepatic adenoma (red arrow).

Cross-sectional imaging showed an indeterminate, wedge-like geographic focus of severe hypoattenuation with internal hyperattenuating foci in the subcapsular right hepatic lobe, with periportal edema and no convincing evidence of background liver disease, suggestive of hepatic hemangioma or hepatic adenoma (Figure 2). There was no evidence of biliary obstruction.

**Figure 3:**
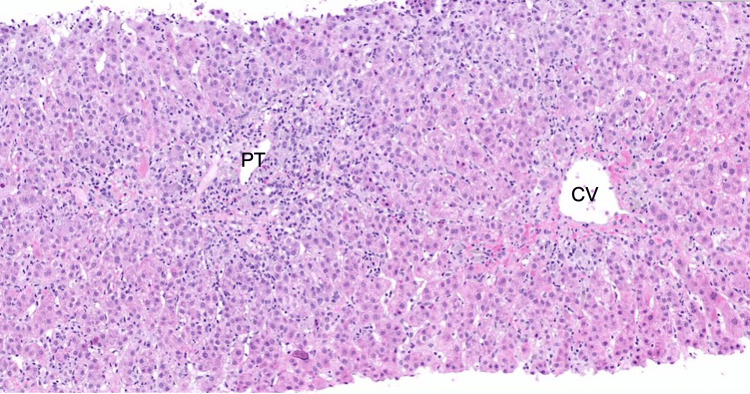
Liver biopsy showed active hepatitis with marked interface activity and areas of confluent necrosis consistent with autoimmune hepatitis (AIH). A representative portal tract (PT) and central vein (CV) are illustrated.

The patient underwent further workup with a liver biopsy, which demonstrated moderate to severe active hepatitis with focal areas of confluent necrosis, moderate lymphoplasmacytic portal inflammation with hepatocyte rosettes and rare lobular plasma cells, and periportal fibrosis with focal bridging, consistent with autoimmune hepatitis (Figure 3).

The patient was histologically diagnosed with seronegative autoimmune hepatitis based on a Modified Hepatitis Activity Index (mHAI) of 15 out of 18 and a simplified diagnostic criteria (SDC) of four out of eight.

The patient was started on prednisone with an improvement of her liver chemistry and symptoms. She was subsequently discharged with plans for continued outpatient follow-up.

## Discussion

Here, we present a patient diagnosed with seronegative autoimmune hepatitis secondary to the COVID-19 infection. COVID-19 manifesting as AIH was first reported in India, where a patient presented with right upper quadrant pain, jaundice, elevated liver enzymes, and a high serum IgG level. Given high suspicion for AIH, the patient was empirically treated with steroids. He did not undergo a liver biopsy due to complications of acute respiratory distress syndrome (ARDS) [3]. In comparison, our patient had normal IgG levels. To date, there are a handful of case reports linking autoimmune hepatitis and COVID-19 infection with over 25 cases of autoimmune hepatitis following vaccination with COVID-19 messenger ribonucleotide (mRNA) vaccines [5]. To our knowledge, this is one of the rare cases of seronegative autoimmune hepatitis following the COVID-19 infection.

AIH is typically diagnosed by the detection of autoantibodies and histologic findings. Typical antibodies include ANA and anti-smooth muscle antibody (type 1 AIH), and anti-liver/kidney microsomal-1 and anti-liver cytosolic antigen type antibody (type 2 AIH) [7]. The pathognomonic histologic feature of autoimmune hepatitis is plasma cell-rich lymphocyte-predominant hepatitis localized to the interface zone. Confluent necrosis is associated with worse outcomes [8,9]. The clinical presentation of AIH can range from asymptomatic to acute liver failure [5].

Seronegative hepatitis comprises about 10% of autoimmune hepatitis cases and may be challenging to diagnose due to a lack of specific serologic tests [2,8]. The revised conventional diagnostic criteria and SDC are often used to aid with diagnosis [8]. SDC is highly predictable with over 90% specificity and sensitivity [8].

Standard treatment of AIH includes steroids and immunologic medications such as azathioprine, which is shown to be effective in 80-90% of the patients. Steroid therapy is typically used to induce remission, with an eventual transition to azathioprine for long-term maintenance of remission [7]. Immunosuppressive treatment has not been associated with worse outcomes in patients with AIH affected by COVID-19, and patients typically respond very well to therapy [4].

There are other reported cases of autoimmune diseases following the COVID-19 infection, including Guillain-Barre syndrome, autoimmune thyroid disease, inflammatory bowel disease, etc [9,10]. There are robust studies from multiple specialties examining this relationship, including a group of rheumatologists who found as many as 1928 publications regarding the new-onset systemic and rheumatic autoimmune diseases related to COVID-19. The mechanism of transmission of COVID-19 to an individual is thought to be mediated through angiotensin-converting enzyme 2 (ACE2) receptors in the lungs, where the spike glycoprotein of the severe acute respiratory syndrome coronavirus 2 (SARS-CoV-2) attaches to enable viral entry into the cells, leading to viral replication and spread throughout the body [11]. Coincidentally, the intestinal and cholangiocytes also highly express ACE2 receptors. It is interesting to note that the hepatocellular pattern of injury is more common in hepatic injury related to COVID-19 infections, although ACE2 receptors are only expressed in about 2.6% of hepatocytes [12]. Direct viral cytotoxicity from the binding has been hypothesized to cause such damage [13].

## Conclusions

The association between COVID-19 and autoimmune hepatitis is now more well-known compared to when the virus first emerged. However, when the initial diagnostic autoimmune workup is unrevealing, it is possible to overlook the possibility of seronegative autoimmune hepatitis (SAH). Our case demonstrates the need for clinicians to consider immune-mediated liver injury as a potential cause for elevation in liver enzymes associated with COVID-19 infection, even in the absence of typical AIH antibodies. This consideration could result in earlier treatment of the condition and lead to better overall outcomes for patients. However, further research is needed to gain a better understanding of the causative relationship between COVID-19 and AIH.

## References

[1] Jothimani D, Venugopal R, Abedin MF, Kaliamoorthy I, Rela M (2020). COVID-19 and the liver. J Hepatol.

[2] Wang X, Lei J, Li Z, Yan L (2021). Potential effects of coronaviruses on the liver: an update. Front Med (Lausanne).

[3] Kulkarni AV, Vasireddy S, Sharma M, Reddy ND, Padaki NR (2022). COVID-19 masquerading as autoimmune hepatitis (AIH) flare-the first report. J Clin Exp Hepatol.

[4] Efe C, Dhanasekaran R, Lammert C (2021). Outcome of COVID-19 in patients with autoimmune hepatitis: an international multicenter study. Hepatology.

[5] Zheng H, Zhang T, Xu Y, Lu X, Sang X (2022). Autoimmune hepatitis after COVID-19 vaccination. Front Immunol.

[6] Washington MK (2007). Autoimmune liver disease: overlap and outliers. Mod Pathol.

[7] Terziroli Beretta-Piccoli B, Mieli-Vergani G, Vergani D (2022). Autoimmmune hepatitis. Cell Mol Immunol.

[8] Sherigar JM, Yavgeniy A, Guss D, Ngo N, Mohanty S (2017). Seronegative autoimmune hepatitis a clinically challenging difficult diagnosis. Case Rep Med.

[9] Senthamizhselvan K, Ramalingam R, Mohan P, Kavadichanda C, Badhe B, Hamide A (2021). De Novo Crohn's disease triggered after COVID-19: is COVID-19 more than an infectious disease?. ACG Case Rep J.

[10] Yazdanpanah N, Rezaei N (2022). Autoimmune complications of COVID-19. J Med Virol.

[11] Guo YR, Cao QD, Hong ZS (2020). The origin, transmission and clinical therapies on coronavirus disease 2019 (COVID-19) outbreak-an update on the status. Mil Med Res.

[12] Groff A, Kavanaugh M, Ramgobin D, McClafferty B, Aggarwal CS, Golamari R, Jain R (2021). Gastrointestinal manifestations of COVID-19: a review of what we know. Ochsner J.

[13] Musa S (2020). Hepatic and gastrointestinal involvement in coronavirus disease 2019 (COVID-19): what do we know till now?. Arab J Gastroenterol.

